# Wound Botulism Among Persons Who Inject Black Tar Heroin in New Mexico, 2016

**DOI:** 10.3389/fpubh.2021.744179

**Published:** 2021-12-16

**Authors:** Nicole Middaugh, Leslie Edwards, Kevin Chatham-Stephens, D. Fermin Arguello

**Affiliations:** ^1^Centers for Disease Control and Prevention, Atlanta, GA, United States; ^2^New Mexico Department of Health, Santa Fe, NM, United States

**Keywords:** wound, botulism, heroin, black tar heroin, outbreak, intubation

## Abstract

Outbreaks of wound botulism are rare, but clinicians and health departments should maintain suspicion for signs, symptoms, and risk factors of wound botulism among persons who inject drugs in order to initiate treatment quickly. This report describes an outbreak of three wound botulism cases among persons in two adjacent counties who injected drugs. Provisional information about these cases was previously published in the CDC National Botulism Surveillance Summary. All three cases in this outbreak were laboratory-confirmed, including one case with detection of botulinum toxin type A in a wound culture sample taken 43 days after last possible heroin exposure. Findings highlight the delay in diagnosis which led to prolonged hospitalization and the persistence of botulinum toxin in one patient.

## Introduction

Botulism is a potentially fatal illness caused by a neurotoxin produced most frequently by the bacterium *Clostridium botulinum*. There are six different types of botulism, distinguished by the way in which neurotoxin exposure occurs—foodborne, infant, wound, adult intestinal colonization, iatrogenic, and inhalational. In 2017, 182 laboratory-confirmed botulism cases were reported in the United States (1). Botulism typically manifests with symmetrical descending paralysis and, if untreated, the illness can lead to respiratory failure and death. *C. botulinum* is ubiquitous in soil and produces seven botulinum toxins (A–G). Human illnesses are caused by types A, B, E and, rarely, F (2).

Wound botulism is caused by contamination of a wound with *C. botulinum* spores. Under anaerobic conditions present in a wound, the spores germinate and produce botulinum toxin. Wound botulism in the United States occurs mostly among persons who inject black tar heroin (BTH) subcutaneously or intradermally (i.e., skin popping) (3). Typically, wound botulism accounts for <10% of all U.S. botulism cases annually, and most wound botulism cases are reported from California (1). Table 1 includes epidemiologic information about outbreaks of wound botulism linked to injection drug use. An increase in wound botulism cases among persons who inject drugs (PWID) that used BTH was reported in California from 1988 to 1998 (3). From 2002 to 2018, 376 laboratory confirmed wound botulism cases in the U.S. were among PWID and 10% of cases reported contacts with other cases (1). In 2008, a wound botulism outbreak among six PWID that used heroin was reported in Dublin, Ireland (4). There were no epidemiological links among cases in this outbreak and only one case was laboratory confirmed due to delayed suspicion of botulism among hospital clinicians and challenges with specimen collection and testing. In 2009, a wound botulism outbreak among six PWID that used heroin was reported in North Rhine, Germany (5). Mouse bioassay testing was used to confirm four cases. In late 2013, a cluster of six wound botulism cases among PWID that used heroin was reported in Norway (6). Two cases knew one another however none reported sharing drugs with other cases. From 2014 to 2015, an outbreak of 25 cases of wound botulism among PWID that used heroin was reported in Glasgow, Scotland (7). One married couple was identified among cases; however, no other social links were reported among cases. These outbreaks highlight the geographic variability of wound botulism outbreaks among PWID although it remains challenging to understand the connection among cases and the precise aspects of the injection drug use process such as contamination of drugs and or drug paraphernalia that led to botulism transmission.

**Table 1:** Wound botulism outbreaks among persons who inject drugs (PWID).

**Year(s)**	**Location**	Number of cases*	**Number of lab confirmed cases**	**Botulism toxin identified**	**Botulism testing types used**	**Drug(s) used**	**Reference**
1988–1998	California, USA	105	105	A (most cases)	MBA	BTH	Werner
2005	Germany	16	4	B	Culture, PCR, PFGE, MBA	Heroin	Schroeter
2008	Dublin, Ireland	6	1	B	MBA	Heroin	Barry
2013	Norway	6	4	Not listed	MBA	Heroin	Macdonald
2017	Scotland	25	9	B	MBA, culture, PCR	Heroin	Martin
2017–2018	California, USA	9	8	A	MBA, MS	BTH and Heroin	Peak

*BTH, black tar heroin; MBA, mouse bioassay; MS, mass spectrometry; PCR, polymerase chain reaction; USA, United States of America*.

This report describes an outbreak of wound botulism among three epidemiologically linked persons that injected drugs in New Mexico in 2016. From 1997 to 2015, four confirmed cases of wound botulism were reported in New Mexico, with three cases linked to injection drug use and one following a motor vehicle accident. This investigation highlights the need for enhanced surveillance for wound botulism among close contacts of persons who inject drugs and the importance of health alerts to the medical community to raise clinicians' awareness of wound botulism.

## Methods

During June–July 2016, the New Mexico Department of Health (NMDOH) investigated an outbreak of three wound botulism cases among persons who injected drugs in two neighboring counties in New Mexico. Patients and family members were interviewed by NMDOH in order to collect information about foods eaten and injection drug use in the 3 days prior to illness. Hospital clinicians consulted with NMDOH and with the Centers for Disease Control and Prevention (CDC) Botulism Clinical Consultation Service in order to determine whether treatment with heptavalent botulism antitoxin (BAT) was appropriate for each patient with suspected botulism (8). The CDC operates a botulism clinical consultation service 24 h a day/7 days a week that hospital based clinicians and their respective state health departments can use to obtain BAT. CDC Quarantine Station Officers coordinated shipment of BAT with hospital pharmacists. In adults, BAT is administered using intravenous infusion beginning at an infusion rate of 0.5 mL/min and may be increased to an 2 mL/min infusion rate if the medication is well-tolerated. Infusion in completed in 3.5–6.7 h depending on the rate of infusion used. Clinical specimens were tested for botulinum toxin using mouse bioassay (9) and EndoPEP-MS mass spectrometry (10). Limited details about this outbreak were previously published in the CDC 2016 National Botulism Surveillance Summary (1). CDC reviewed this report for human subjects' protection and deemed it not to constitute research.

## Results

On July 1, 2016, Patient 1 presented to the emergency department with shortness of breath, dysphagia and double vision which began on June 29. The patient experienced respiratory arrest, requiring intubation and mechanical ventilation, and was immediately transferred to a tertiary hospital where wound botulism was suspected based on clinical presentation, history of skin-popping black tar heroin, and presence of a right hip abscess. The patient reported multiple occasions of skin-popping in the days before onset of symptoms. After evaluating the patient, the consulting neurologist contacted the NMDOH to request BAT. NMDOH contacted CDC on July 2, 2016, for consultation and to arrange BAT shipment and specimen testing. The patient received BAT and penicillin on July 2, 2016. Botulinum toxin type A was detected by mouse bioassay in serum collected on July 1. The patient was transferred to a long-term care facility until they were extubated, and subsequently required inpatient physical rehabilitation.

NMDOH issued a Health Alert Network (HAN) notification to clinicians throughout New Mexico on July 12, 2016, communicating the potential for *C. botulinum*-contaminated black tar heroin and advising clinicians to consider botulism in patients with neurological signs and symptoms, particularly those who skin-pop black tar heroin. NMDOH collaborated with the New Mexico Harm Reduction Program to distribute information to participants of New Mexico's needle exchange program, urging persons with signs and symptoms of botulism to seek immediate medical attention.

On July 14, a regional emergency department physician notified NMDOH of a new patient with suspected botulism (“Patient 2”) who presented with blurry vision, dysphagia, dysarthria, neck weakness and extremity weakness which began on July 11. The physician had read the HAN notification and obtained the patient's history, including skin-popping black tar heroin. The patient reported being an intimate partner of Patient 1 and had recently shared heroin with the patient. Because of their neurologic symptoms, connection to Patient 1 and black tar heroin use, Patient 2 was transferred to a tertiary hospital where specimens were collected for botulism testing. NMDOH immediately contacted CDC for clinical consultation and to obtain BAT. The patient received BAT 9 h after presenting to the regional emergency department. Botulinum toxin type A was detected by EndoPEP-MS testing in serum collected on July 14. Patient 2 was discharged home 4 days after receiving BAT, never requiring intubation.

On June 23, 2016, Patient 3 presented to a local hospital complaining of a spider bite, dysphagia, diplopia, lip swelling and tongue swelling with onset the same day. They had an indurated area on the right buttock with reddish-purple discoloration but no visible abscesses and was discharged home with antibiotics. On June 24, Patient 3 was found unresponsive at home and was taken to another hospital by emergency medical services and was intubated shortly after arrival due to respiratory failure initially attributed to illicit substance toxicity. On July 4, 11 days after onset, Patient 3 was transferred to a tertiary hospital after their inability to wean from mechanical ventilation and significant extremity weakness. After an extensive workup that included an inconclusive nerve conduction study and needle electromyography, but did not include a CT scan for abscesses, the patient was diagnosed with Miller-Fischer variant Guillain-Barré Syndrome. Botulism was on the differential diagnosis; however, botulinum toxin testing was not conducted. The patient was treated with intravenous immunoglobulin (IVIG) and transferred to a long-term care facility 5 days later.

NMDOH discussed the possibility of the patient having botulism with the long-term care facility (LTCF) physician. The LTCF physician was also caring for Patient 1 and suspected that Patient 3 also had botulism, not Guillain-Barré Syndrome. On July 14, NMDOH interviewed Patient 3 as a potential third case in the outbreak, because of their continued symptoms and failure to improve after IVIG. Patient 3 reported skin-popping black tar heroin on June 24 and that they knew the index patient, Patient, 1 but that they had not shared drugs or drug paraphernalia. NMDOH worked with the physicians to obtain serum samples previously drawn on July 4 (12 days after onset) and newly drawn on July 17 (25 days after onset) for botulinum toxin testing. On July 29, botulinum toxin type A was detected by EndoPEP-MS in serum collected on July 4. Because more than 3 weeks had passed since that serum had been collected, and it was unclear if the patient still had circulating botulinum toxin, the LTCF physician opted to wait to request BAT, pending test results of the July 17 drawn serum. On August 5, botulinum toxin type A was detected in the July 17 serum tested by EndoPEP-MS. The patient's neurological signs and symptoms had still not improved; therefore, a CT scan of the hips was conducted to identify a persistent source of toxin. The CT revealed two occult abscesses deep in the patient's right and left buttocks. On August 6, the right-side abscess was drained, serum and wound cultures were obtained, and BAT was administered. Wound cultures tested positive for botulinum toxin type A-−43 days after symptom onset and the last possible exposure to black tar heroin. Botulinum toxin type A was not detected by mouse bioassay in serum drawn on August 6, which was obtained before BAT was administered. Patient 3 required mechanical ventilation for 110 days. They were eventually transferred to an inpatient physical rehabilitation facility. Table 2 lists the onset date, time from presentation to BAT administration, duration of mechanical ventilation, and outcome for the three patients.

**Table 2:** Time to botulism antitoxin (BAT) administration, duration of mechanical ventilation, and outcome among wound botulism patients (*N* = 3)^a^.

	**Onset date**	**Time from illness onset date to mechanical ventilation**	**Time from illness onset date to botulism antitoxin**	**Laboratory confirmed**	**Botulism testing types used**	**Botulism toxin identified**	**Duration of mechanical ventilation**	**Patient outcome**
Patient 1^b^	6/29/2016	2 days	5 days	Yes	MBA	A	30 days	Survived
Patient 2	7/11/2016	n/a	9 h	Yes	MS	A	Not required	Survived
Patient 3^b^	6/23/2016	1 day	43 days	Yes	MS, wound culture	A	110 days	Survived

a *Time from exposure to onset of symptoms is unavailable due to multiple instances of injection drug use prior to onset of signs and symptoms*.

b *Required inpatient physical rehabilitation*.

*MBA, mouse bioassay; MS, mass spectrometry*.

## Discussion

NMDOH investigated an outbreak of three laboratory-confirmed, epidemiologically linked, type A wound botulism cases among persons who injected black tar heroin. The persons presented to emergency departments with neurologic signs and symptoms associated with botulism (e.g., diplopia, dysphagia). Patient 2 was promptly diagnosed with botulism and treated with BAT within 9 h; this patient did not develop respiratory compromise and hospital discharge occurred within a few days. Wound botulism was not considered during patient 1 and patient 3's initial emergency department evaluations and consequently wound care and BAT administration were delayed. Both suffered respiratory arrest and required intubation and mechanical ventilation for weeks or months. All three patients engaged in behaviors associated with wound botulism, including skin-popping and using black tar heroin (3, 11, 12). Two patients shared drugs and needles, and each had an occult or visible abscess.

Wound botulism diagnosis among patients who inject drugs continues to be challenging. Since this outbreak was reported in 2016, a wound botulism outbreak among nine persons who injected black tar heroin and/or heroin was reported in San Diego, California (13). Mechanical ventilation was required for 6 (67%) patients and the median duration of hospitalization was 15 days (range: 9–67 days). There was a delay in botulism diagnosis for four (44%) of patients who were initially diagnosed with drug intoxication. Both the New Mexico outbreak and the San Diego outbreak demonstrate the need for increased awareness among hospital clinicians about the possibility of wound botulism among persons who inject drugs.

BAT is a well-tolerated medication with a low incidence of adverse events. Among 249 patients treated with BAT, the most frequently reported adverse events included fever (4%), rash (2%), chills, agitation, edema or nausea (1% each) (14). Mild serum sickness was reported in one patient 11 days after receiving BAT. Patients treated with BAT should be monitoring for signs of hypersensitivity during BAT infusion and afterwards for adverse events (8). In this outbreak, there was an association between the duration of ventilator use and the amount of time between onset of symptoms and BAT receipt. This observation is consistent with prior reports that earlier treatment with botulism antitoxin is linked to better clinical outcomes including decreased duration of hospitalization (7, 15) and decreased mortality (16).

From 2002 to 2018 in the United States, 95% of lab confirmed wound botulism cases were reported among PWID (17). Traumatic injury was reported among 3% of cases with the remainder of cases having no identified risk factor for wound botulism. Traumatic injuries reported among cases include motor vehicle accident, gunshot wound, or other injury resulting in an open fracture. Wound botulism is a severe and often life-threatening illness that requires rapid identification and proper treatment, including identifying and debriding abscesses, providing antimicrobial therapy when indicated, and administering BAT as soon as possible. Among 94 patients with wound botulism from injecting drugs, Werner et al. (3) reported that 14 (15%) patients had wounds that did not appear grossly infected. Detailed examination or injection site imaging might be needed to identify deep abscesses, as in Patient 3. The implementation of new botulism testing including EndoPEP-mass spectrometry has been helpful in decreasing the amount of time needed to complete botulism clinical testing, although mass spectrometry is available in very few public health laboratories. In many ways wound botulism may be more challenging to diagnose than foodborne botulism due to the sensitive nature of exposures among PWID. Wound botulism remains a challenge to rapidly diagnose and treat as patients may be hesitant to seek medical care and to report use of illegal drugs including heroin. Skilled interviewers are needed to help gather information about exposures among PWID. An additional challenge in diagnosing wound botulism among PWID is that certain signs and symptoms of botulism may be misdiagnosed as opiate overdose. Clinicians suspecting botulism in a patient should immediately call the emergency telephone number at their state health department to request a clinical consultation for botulism. The CDC Botulism Clinical Consultation Program is available 24 h a day, 7 days a week with staff available to help consult with hospital-based clinicians and health department staff in order to facilitate treatment with BAT and botulism testing.

## Conclusions

Outbreaks of wound botulism are rare, but clinicians and health departments should maintain suspicion for signs, symptoms, and risk factors of wound botulism among persons who inject drugs—especially among persons who skin-pop black tar heroin—to initiate treatment quickly. Minimizing the time from presentation to abscess debridement and to BAT administration, has been shown to reduce the need for mechanical ventilation and decrease the duration of intensive care (18, 19). In this outbreak, we detected botulinum toxin type A in a wound culture 43 days after last possible heroin exposure. Early detection and debridement of abscesses, use of imaging, and treatment with antibiotics and BAT are essential to prevent ongoing toxin production. Communication between clinicians, health departments, and CDC is key to rapid identification of cases, especially in outbreaks.

## Author Contributions

All authors listed have made a substantial, direct, and intellectual contribution to the work and approved it for publication.

## Funding

This work was provided by the New Mexico Department of Health and the Centers for Disease Control and Prevention.

## Conflict of Interest

The authors declare that the research was conducted in the absence of any commercial or financial relationships that could be construed as a potential conflict of interest.

## Publisher's Note

All claims expressed in this article are solely those of the authors and do not necessarily represent those of their affiliated organizations, or those of the publisher, the editors and the reviewers. Any product that may be evaluated in this article, or claim that may be made by its manufacturer, is not guaranteed or endorsed by the publisher.
